# Supplementary material for: Assessment of Phenotype Microarray plates for rapid and high-throughput analysis of collateral sensitivity networks
Source: PLoS One. 2019 Dec 18;14(12):e0219879. doi: 10.1371/journal.pone.0219879 (PMC6919586; doi:10.1371/journal.pone.0219879)
Supplement: S5 Fig — Scores are an average of two biological replicates, with 8 representing maximum relative resistance around the exterior of the radar and 0 representing complete sensitivity at the centre. Clinical isolates are in shades of blue and green. Laboratory strains S. aureus ATCC 25923 and 25923evo are orange and red respectively. (PDF) [file pone.0219879.s005.pdf]

Assessment of Phenotype Microarray plates for rapid and high-throughput analysis of collateral sensitivity networks

Elsie J. Dunkley, James D. Chalmers, Stephanie Cho, Thomas J. Finn, Wayne M. Patrick

Supporting Information

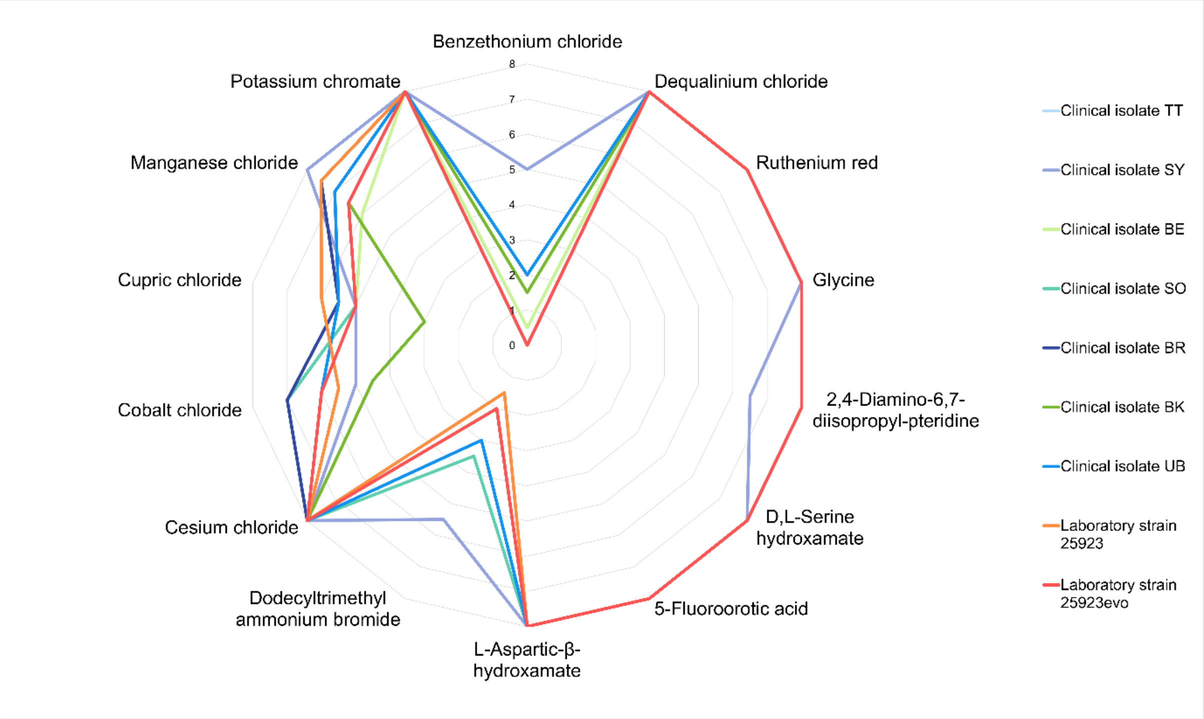

S5 Fig. Phenotype Microarray scores for nine *S. aureus* strains in the presence of antiseptics, disinfectants, metal ions, etc. Scores are an average of two biological replicates, with 8 representing maximum relative resistance around the exterior of the radar and 0 representing complete sensitivity at the centre. Clinical isolates are in shades of blue and green. Laboratory strains *S. aureus* ATCC 25923 and 25923evo are orange and red respectively.
