# Supplementary material for: Assessment of Phenotype Microarray plates for rapid and high-throughput analysis of collateral sensitivity networks
Source: PLoS One. 2019 Dec 18;14(12):e0219879. doi: 10.1371/journal.pone.0219879 (PMC6919586; doi:10.1371/journal.pone.0219879)
Supplement: S1 Table — (PDF) [file pone.0219879.s006.pdf]

Assessment of Phenotype Microarray plates for rapid and high-throughput analysis of collateral sensitivity networks

Elsie J. Dunkley, James D. Chalmers, Stephanie Cho, Thomas J. Finn, Wayne M. Patrick

Supporting Information

S1 Table. Full list of antimicrobial compounds tested in PM plates 11, 12 and 13, grouped according to mode of action.

Cell wall-acting antibiotics:
Amoxicillin
Cloxacillin
Nafcillin
Penicillin G
Oxacillin
Carbenicillin
Ampicillin
Azlocillin
Cefazolin
Ceftriaxone
Cefuroxime
Moxalactam
Cephalothin
Vancomycin
Polymyxin B
Colistin

Protein synthesis inhibitors:
Amikacin
Capreomycin
Gentamicin
Kanamycin
Neomycin
Paromomycin
Sisomicin
Tobramycin
Spectinomycin
Geneticin (G418)
Chlortetracycline
Demeclocycline
Minocycline
Tetracycline
Penimepicycline
Doxycycline
Rolitetracycline
Chloramphenicol
Erythromycin
Spiramycin
Tylosin
Lincomycin

Inhibitors of nucleic acid synthesis:
Sulfamethazine
Sulfadiazine
Sulfathiazole
Sulfamethoxazole
Nalidixic acid
Oxolinic acid
Enoxacin
Lomefloxacin
Ofloxacin
Novobiocin
Rifampicin

Repurposed cancer and anti-psychotic drugs:
Bleomycin
6-Mercaptopurine
Cytosine-1-β-D-arabino-furanoside
5-Fluorouracil
Potassium tellurite
Thallium (I) acetate

Antiseptics, disinfectants, metal ions, *etc*:
Benzethonium chloride
Dequalinium chloride
Ruthenium red
Glycine
2,2'-Dipyridyl
2,4-Diamino-6,7-diisopropyl-pteridine
D,L-Serine hydroxamate
5-Fluoroorotic acid
L-Aspartic-β-hydroxamate
Dodecyltrimethyl ammonium bromide
Cesium chloride
Cobalt chloride
Cupric chloride
Manganese chloride
Nickel chloride
Potassium chromate
